# Epidemiology of Hepatitis E Virus in China: Results from the Third National Viral Hepatitis Prevalence Survey, 2005–2006

**DOI:** 10.1371/journal.pone.0110837

**Published:** 2014-10-31

**Authors:** Zhiyuan Jia, Yao Yi, Jianhua Liu, Jingyuan Cao, Yong Zhang, Ruiguang Tian, Tao Yu, Hao Wang, Xinying Wang, Qiudong Su, Wenting Zhou, Fuqiang Cui, Xiaofeng Liang, Shengli Bi

**Affiliations:** 1 National Institute of Virology Disease Control and Prevention, Chinese Center for Disease Control and Prevention, Beijing, China; 2 Guangzhou Center for Disease Control and Prevention, Guangzhou, China; 3 Chinese Center for Disease Control and Prevention, Beijing, China; Virginia Polytechnic Institute and State University, United States of America

## Abstract

In China, hepatitis E virus (HEV) is prevalent and causes disease, but its epidemiological profile is not well understood. We used a commercial enzyme-linked immunosorbent assay to detect total antibodies to hepatitis E virus in 15,862 serum samples collected during the Third National Viral Hepatitis Prevalence Survey. The results were analyzed to calculate estimates of HEV seroprevalence and to examine the effects of some putative risk factors. The seroprevalence of HEV in the general Chinese population during the period from 2005 through 2006 was 23.46% (95% confidence interval [CI], 18.41%–28.50%). The farming population, the age group of 15–60 year olds, and those living in the Midwest or Mideast region and in Xinjiang province had the highest seroprevalence estimates. The prevalence of HEV is high in China. The seroprevalence rate of HEV shows an unbalanced distribution among areas with different geographic location and economic development levels. The characteristics of the distribution associated may be due to the route of HEV transmission (via contaminated water or animal reservoirs). Within the same region, the seroprevalence of HEV is generally increased with age.

## Introduction

Hepatitis E is an endemic disease in many regions of the world, and hepatitis E virus is the causative agent [1]. According to World Health Organization, there are approximately 20 million incident HEV infections every year, with over 3 million acute cases of hepatitis E and 56,600 deaths related to hepatitis E worldwide [2], [3].

In endemic regions, hepatitis E is responsible for many water-borne epidemics and sporadic cases of acute hepatitis. In these areas, infection is generally transmitted through the fecal–oral route via contaminated water. Less frequent routes of transmission include contaminated food, transfusion of infected blood products and vertical (materno-fetal) transmission. In developed countries, hepatitis E was initially found to occur among travelers to disease-endemic regions [3], [4]. However, an increasing number of autochthonous cases have been identified over the last decade and more evidence for a zoonotic reservoir has been uncovered [5]–[7]. This has led to a global resurgence of interest in this disease.

China is usually judged to be an endemic area for hepatitis E. With the availability of reliable HEV diagnostics and national hepatitis A vaccination campaigns, the reported number of acute hepatitis E cases in China has increased rapidly in recent years, surpassing hepatitis A in some areas.

A high prevalence of antibodies to HEV (anti-HEV) among healthy individuals has been reported in some regions of China. However, these surveys were generally of limited geographic scope [8]–[13]. To estimate the prevalence of HEV infection in the general population of China, we tested a nationally representative serosample for anti-HEV using a commercial diagnostic kit with a highly sensitive and specific enzyme-linked immunosorbent assay. We documented the HEV seroprevalence in the general population of China and examined associations between HEV seropositivity and putative risk factors.

## Methods

Study procedures were approved by the Chinese CDC Ethics committee, and all study work was performed in accordance with the national ethics regulations. Written (signature or thumbprint) informed consent was obtained from all adult participants, supported by the signature or thumbprint of a second adult witness to the consent process; parental consent was sought for children, accompanied by age-appropriate assent. Participants testing positive for antibodies to HEV were informed of their status and counseled using ethics committee-approved messages.

### Study population

From 2005 through 2006, the Third National Viral Hepatitis Prevalence Survey (NVHPS III) (not including Taiwan) was conducted by the Chinese Center for Disease Control and Prevention (China CDC). NVHPS III was a cross-sectional study of the Chinese population that was designed to provide national statistics on the prevalence of major viral hepatitis. The survey data will be used in epidemiological studies and health sciences research, in order to help develop a public health policy for viral hepatitis, direct and design hepatitis health programs and services, and expand the knowledge on viral hepatitis in the country [14].

The study population comprised residents aged 1–59 years old who lived in 160 disease surveillance points (DSP) in 31 provinces of China. The DSP were selected by China CDC to be representative of the population of China. In the DSP, the demographics, economic conditions and the environment of the population are not statistically different from those of the whole country. Overall, 81,775 serosamples were eligible for data analysis and stored at −40°C in our laboratory.

To detect anti-HEV, we selected a subsample from the 81,775 serosamples in the NVHPS III. According to the expected hepatitis E prevalence by different age groups (5% at 1 to 10 years, 10% at 11 to 15 years, 20% at 16 to 20 years, 30% at 21 to 30 years, 40% at 31 to 40 years, 50% at 41 to 59 years), the sample size for each age group was calculated using the formula: n = (Z_α/2_)^2^P(100-P)/ε^2^ and the total desired sample size of the subsample was 14,150. To increase the sample size appropriately, the adjusted sample size (which allows for non-detection and was calculated as follow: n′ =  (100/f)*N, f = 85, N = 14150) was 16,647. Each subsample was randomly selected from the serosamples by use of stratified sampling according to the participant's sex and age.

### Laboratory methods

All serum specimens were tested at the National Hepatitis Laboratory of the Institute for Viral Disease Control and Prevention (IVDC) at the China CDC. We used an MP Diagnostics (MPD) HEV ELISA 4.0 to test the samples for specific HEV antibodies(Batch number AG20131005). The MPD HEV ELISA 4.0 is an enzyme-linked immunosorbent assay intended for the detection of total antibodies to hepatitis E virus in human serum or plasma. It utilizes a proprietary recombinant antigen, which is highly conserved among different HEV strains, to detect the presence of specific antibodies, including IgG, IgM and IgA, against HEV. For specimens with inconsistent results, the MPD HEV ELISA 4.0 was used for confirmatory testing.

### Statistical methods

Survey weights were used to calculate point estimates: w*_p_* was the weight for a township in the NVHPS III, w*_s|p_* the weight for a village within the selected township, w*_t|p,s_* the weight for an individual within the selected village, w*_q|p,s,t_* the weight for the subsample associated with the NVHPS III, and ww*_adj_* was the calibration factor for each subsample that adjusts the sample age and sex distribution to agree with that of the Chinese population as a whole. The final weight for person *i* is:
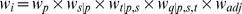



We used the Taylor series linearization method to calculate variances of the point estimates, and an approximate 95% CI (confidence interval) for each estimate was constructed. For each pair of variables, if the 95% CI did not overlap the result was considered to be statistically significant. A logistic regression model for complex survey data was used to examine associations between HEV prevalence and putative risk factors. All statistical analyses were performed using PROC SURVEYFREQ and PROC SURVEYLOGISTIC in SAS software (version 9.3, SAS Institute).

## Results

Among 16,647 selected samples from NVHPS III study participants at 1–59 years of age, the 15,852 samples were eligible for data analysis. Statistical analysis of results from the 15,852 eligible samples estimated the seroprevalence of HEV in the general Chinese population from 2005 through 2006 was to be 23.46% (95% confidence interval [CI], 18.41%–28.50%). Seroprevalence estimates specific to sex, occupation, race/ethnicity, geographic region and age group are shown in Table 1. As seen in Table 1, the seroprevalence of HEV among individuals born in Zhuang and Uigur was significantly higher than for other ethnic groups. The seroprevalence in farmers was significantly higher than for other occupations (34.38%, 95% CI 28.43–40.32). The age group of 15–60 year olds had a significantly higher seroprevalence than 1–14 year olds (28.04%, 95%CI 22.08–33.99 to 6.77, 95%CI 5.13–8.41).

**Table 1:** Adjusted Seroprevalence of HEV antibodies in the Chinese population in the national sample, 2005–2006, by gender, occupation, ethnic origin, geographic area, economic development areas and age groups.

Variable	Samples tested, no.	Weighted Positive for anti-HEV	
		%	(95% CI)
sex			
Female	7856	22.24	14.31–30.16
Male	7996	24.64	19.98–29.30
Age group(yrs)			
1–14	11790	6.77	5.13–8.41
15–60	4062	28.04	22.08–33.99
Occupation			
Children	5077	4.4	2.85–5.96
Student	7795	8.96	6.65–11.26
Farmer	1807	34.38	28.43–40.32
Worker	383	24.55	15.97–33.13
others	790	19.96	12.28–27.64
Ethnicity			
Han	13691	22.28	16.93–27.64
Mongolian	125	10.37	0–25.06
Tibetan	429	17.99	9.26–26.72
Uigur	272	46.61	39.84–53.38
Zhuang	84	72.02	53.47–90.56
Hui	238	21.15	0–45.25
others	981	31.14	15.33–46.95
Region			
Eastern	5196	18.68	14.36–22.99
Middle	5298	19.12	14.70–23.55
Western	5358	32.38	17.25–47.50
Urban/Rural			
Urban	7842	22.77	19.31–26.22
Rural	8010	23.68	17.10–30.26
Total	15852	23.46	18.41–28.50

**Figure 1:**
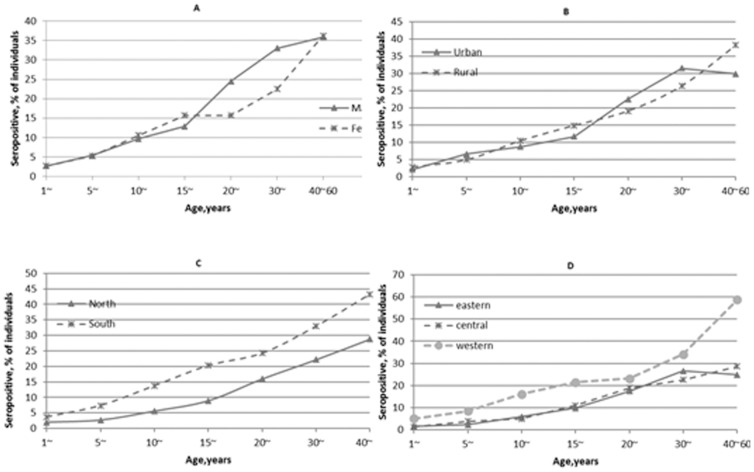
Adjusted seroprevalence of HEV antibodies in the Chinese population by age group for individuals, with regard to sex(A), urban/rural habitation(B), geographic area(C)and economic development area(D). (Western provinces include: Chongqing, Gansu, Guangxi, Guizhou, Inner Mongolia, Ningxia, Qinghai, Shanxi, Sichuan, Tibet, Yunnan and Xinjiang. Central Provinces include: Anhui, Hainan, Hebei, Heilongjiang, Henan, Hubei, Hunan, Jiangxi, Jilin and Shanxi. Eastern provinces include: Beijing, Fujian, Guangdong, Jiangsu, Liaoning, Shandong, Shanghai, Tianjin and Zhejiang. Urban counties are defined as that counties which are capital in prefecture; rural counties are those counties which are not capital of the prefecture.)

The seroprevalence of HEV was generally low among children and increased with age. In males, the increase with age was most marked in 15–29 year olds, and in females this occurred in 20–39 year olds. With regard to geographic area, individuals living in the south had higher regional seroprevalence estimates than in north China. Among the different economic development areas, individuals living in western China had the highest regional seroprevalence estimates, but there were no significant differences among geographic areas and economic development areas (Figure 1).

**Figure 2:**
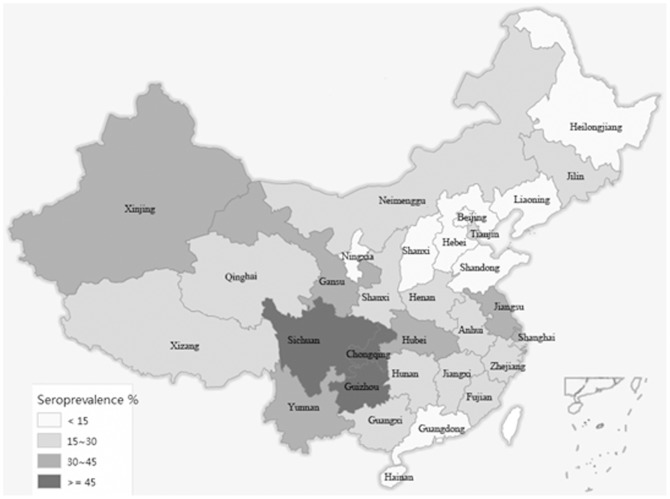
Adjusted seroprevalence of HEV antibodies in the Chinese population by province.

Seven provinces (Sichuan, Guizhou, Yunnan, Xinjiang, Shanghai, Hubei and Jiangsu) had the highest seroprevalence estimates in China. Nine provinces (Gansu, Zhejiang, Henan, etc.) had intermediate estimates and the other provinces had lower HEV seroprevalence estimates (Figure 2).

Having hepatitis A virus (HAV) seropositivity (OR, 1.62 [95% CI, 1.22–2.16]), the seroprevalence in farmers (OR, 2.78 [95% CI, 1.47–5.30]), the age group of 15–60 year olds(OR, 3.86 [95% CI, 2.58–5.78]) and individual living in western China(OR, 2.85 [95%CI, 1.18–6.88]) were associated with significantly higher odds of HEV seropositivity. No other statistically significant associations were observed between risk factors and HEV seropositivity (Table 2).

**Table 2:** Odds ratios(OR) and 95% confidence intervals (CI) for the associations between different variables and the seroprevalence of HEV antibodies in the Chinese population in 2005–2006, as found in an univariate logistic regression adjusted by age, gender and in a multivariate logistic regression model.

Variable	Samples tested, no. (pos./neg.)	Univariate analysis			Multivariate analysis		
		OR	(95% CI)	P Value	OR	(95% CI)	P Value
HAV antibody (2052 missing)							
No	373/5580	Ref.					
Yes	1047/6799	1.90	1.41–2.55	<0.0001	1.62	1.22–2.16	0.001
HBV HBsag							
No	1552/13937	Ref.					
Yes	73/290	0.65	0.34–1.23	0.19	0.75	0.40–1.40	0.37
Region							
Eastern	380/4816	Ref.					
Middle	365/4924	1.02	0.67–1.55	0.94	0.98	0.63–1.5	0.91
Western	880/4477	2.42	1.14–5.17	0.02	2.85	1.18–6.88	0.02
Urban/Rural							
Urban	746/7096	Ref					
Rural	880/7130	1.09	0.72–1.64	0.69	0.75	0.49–1.12	0.16
Gender							
Female	823/7033	Ref					
Male	802/7194	1.19	0.73–1.95	0.48	1.52	0.95–2.44	0.08
Age group(yrs)							
1–14	694/11096	Ref.					
15–60	931/3131	5.42	4.37–6.72	<0.0001	3.86	2.58–5.78	0.001
Occupation							
Children	173/4904	0.29	0.15–0.58	0.0004	1.78	0.67–4.76	0.25
Student	642/7153	0.53	0.34–0.83	0.005	0.80	0.41–1.57	0.51
Farmer	517/1290	2.08	1.33–3.25	0.001	2.78	1.47–5.30	0.002
Worker	98/285	1.30	0.88–1.94	0.19	1.34	0.88–2.03	0.17
Others	195/595	Ref.					

## Discussion

In Western Europe and many developed countries in the world, hepatitis E infection is relatively infrequent [15]–[22]. However, in a study on 18,000 sera tested in the Third National Health and Nutrition Examination Survey (NHANES III) in United States in 2006, the anti-HEV seropositivity rate was 21% [22]. This conflicts with an estimated annual incidence of 0.7% in the United States [21]. The autochthonous hepatitis E virus that explains some of the HEV seroprevalence observed in that study is attributable to exposure to infected animals (foodborne zoonotic infections or from contact with animals), previous subclinical HEV infection and infections acquired in developing countries. In Asia, Africa, Central America, and other developing countries, hepatitis E occurs both sporadically and as epidemic disease. Published rates of anti-HEV antibody among adults range from 30 to 80% in these areas [23]–[29].

The HEV seroprevalence estimate observed in the current study was 23.46% (95% confidence interval [CI], 18.41%–28.50%). The results suggest that exposure to HEV is common in the general Chinese population. In other reports, anti-HEV seropositive estimates range from 10.77% to 52% in the general population in China [8]–[13], [30]–[36]. The reasons for the varying rates of seropositivity are that these surveys were conducted in different regions, on different study populations and using diagnostic reagents of different sensitivities. In the current study, the result is similar to the prevalence of anti-HEV (21%) reported in a study of hepatitis E virus markers in Chinese people residing in Tokyo, Japan [37]. However, it is higher than the seropositive rate of 17.2% which was estimated in the Second National Viral Hepatitis Prevalence Survey in China in 1992 [32]. Given that the 1992 survey did not include all 31 provinces, the current study can be seen as the first nationwide description of the seroepidemiological characteristics of HEV in China. In other nationwide general population surveys, the seroprevalence of anti-HEV antibodies was 21% in the United States [22], 5.3% in Japan [17], and 1.9% in the Netherlands [15]. However, these studies are difficult to compare because of the differences in assays used. In our study, total antibodies to HEV were detected using the MP Diagnostics HEV ELISA4.0, which is the latest generation enzyme-linked immunosorbent assay produced by MP corporation, and is intended for the detection of total antibodies to hepatitis E virus in human serum or plasma.

In China, most infections are due to HEV genotypes 1 and 4, though HEV genotype 3 has been reported recently [8]–[12]. In 1989, a widespread outbreak resulted in 120,000 acute cases of hepatitis E in Xinjiang Province, northwestern China. The outbreak was caused by HEV genotype 1 [30]. Since then, hepatitis E in China has occurred mainly as sporadic cases and occasional food-borne outbreaks. The predominant circulating genotype is HEV genotype 4, with a few occasional cases involving genotype 1. Hepatitis E virus is principally a water-borne disease and the infection is most often transmitted by contaminated water. The reservoir of HEV responsible for maintaining the disease in a population has not been identified. The reservoir of the virus may be a continuously circulating pool of humans with subclinical HEV infection, although other animals, such as pigs, are a potential reservoir of the virus. This pool of infection may lead to periodic contamination of drinking water supplies and raise the prevalence of HEV in a region. Although all four HEV genotypes infect humans, only genotypes 3 and 4 also infect other animal species, especially swine, which may act as zoonotic reservoirs of infection [4], [23], [38].

In our study, there was no simple geographic division of the HEV prevalence in China, but the HEV prevalence was significantly different among provinces. We found that Sichuan, Guizhou, Yunnan, Hubei, Henan, Zhejiang, Jiangsu and Shanghai had the highest HEV seroprevalence; They are mostly distributed in the Midwest and Mideast regions of China. The provinces of Ningxia, Shanxi and Guangdong had the lowest seroprevalence of HEV. This result was consistent with some other studies in China [8], [33], [36], [39]. Contaminated water and zoonotic reservoirs of infection may play an important role in the distribution of HEV seroprevalence among the provinces in China, although further research is needed to study the relationship between the development of the pig-breeding industry and the high HEV seroprevalence in Sichuan, Guizhou, Yunnan, Henan and Jiangsu provinces, and the relationship of low HEV seroprevalence with religious practices in Ningxia, where most local people are of Hui nationality. In particular, in Shanghai and Xinjiang, where two widespread outbreaks of food-borne and water-borne infections resulted in large numbers of acute cases of HAV and HEV respectively, in 1988 and 1989 [30], [40], [41], there remained a high HEV seroprevalence in the present study.

Hepatitis E infection is acquired from an environmental, human or other animal reservoir via poor general sanitation, contaminated drinking water supplies and lack of attention to personal hygiene. Individuals living in western provinces of China, which remain relatively underdeveloped in terms of economy and social systems, had the highest regional seroprevalence estimates. This result seems to support the above findings, but there was no significant difference in HEV seroprevalence between rural and urban residents in our survey. In addition, high seroprevalence estimates were found in some developed regions, such as Shanghai, Jiangsu and Zhejiang. This is similar to the situation in the USA [22]. As in developed countries [4], [22], a possible reason for this observation is that the predominant circulating genotype in China is HEV genotype 4, with occasional cases involving genotype 1.

The seroprevalence of HEV in a population generally increases with age [15]–[18], [22], [25]. In our study, the seroprevalence of HEV increased throughout the whole age range, the first marked increase in seropositivity occurred at 15 to 29 years in males and 20 to 39 years in females. Thus, HEV infection occurs at all ages but that people over 15 years have higher rates of seropositive to HEV. This contrasts with hepatitis A: the first marked increase in the seroprevalence of HAV occurs at the age of 1 to 10 years and the prevalence does not vary significantly after 20 years of age [2], [14], [32]. Given that this was not a birth cohort study, the difference HEV seroprevalence among age groups represented only the presence of anti-HEV antibodies, not the changes in the prevalence of infection of HEV with age.

Some studies have shown that the rate of hepatitis E seropositivity is significantly higher in males than in females [17], [33]; although some studies show higher rates in females [6], and other studies show no significant difference between male and female populations [15], [28]. The difference in HEV seroprevalence between male and female participants in our study was just below the level of significance. This discrepancy may be related to the different populations studied, and possibly may be related to genotype.

On comparison of different occupations, our results were consistent with those of a study from the UK in which the HEV seroprevalence in the farming population was the highest among all occupations [7]. This may be because the farming community is more likely to have contact with feces from infected animals, or with water or food contaminated by HEV, than those in other occupations. The large difference HEV seropositivity was seen between students and farmers, however this has has less to do with the difference between the occupations than with confounding by age.

We observed a positive association between HAV and HEV seropositivity in a multivariable model adjusted for age and sex. The positive association disagrees with the findings of a study of HEV seroprevalence in the USA [22]. It is possible that the positive association between HAV and HEV seropositivity may be explained by a relationship with age or route of transmission

The prevalence of HEV is high in the general Chinese population. The seroprevalence of HEV shows an unbalanced distribution among geographic areas. The characteristics of the distribution, when associated with the route of HEV transmission, suggest that it is mainly transmitted via contaminated water and animal reservoirs. Within each region, the seroprevalence of HEV generally increases with age. Farming, an age of 15–60 years, HAV seropositivity and living in western China were found to be risk factors for HEV infection.

Given that this was a cross-sectional survey performed from 2005 to 2006, the result should only reflect the seroprevalence of HEV in those year. The situation may have changed since then. Further study is required to shed light on long-term epidemiological trends and the regional distribution of HEV infection in China, including the route of infection, the clinical outcomes and the genotype associated with HEV infection. The first prophylactic vaccine, Hecolin, against hepatitis E virus infection and HEV-associated disease was approved by the Chinese State Food and Drug Administration (SFDA) in December 2011 and is being marketed in China [42]–[44]. This represents a major development in the prevention of HEV infection and may transform public health.
